# Infective Endocarditis and Antimicrobial Timing: A Case for Delay?

**DOI:** 10.1093/ofid/ofaf628

**Published:** 2025-10-07

**Authors:** Elisavet Stavropoulou, Bruno Ledergerber, Nicolas Fourré, Virgile Zimmermann, Jana Epprecht, Nicoleta Ianculescu, Pierre Monney, Georgios Tzimas, Michelle Frank, Laurence Senn, Lars Niclauss, Matthias Kirsch, Mathias Van Hemelrijck, Omer Dzemali, Benoit Guery, Barbara Hasse, Matthaios Papadimitriou-Olivgeris

**Affiliations:** Infectious Diseases Service, Lausanne University Hospital and University of Lausanne, Lausanne, Switzerland; Department of Infectious Diseases and Hospital Epidemiology, University Hospital Zurich and University of Zurich, Zurich, Switzerland; Infectious Diseases Service, Lausanne University Hospital and University of Lausanne, Lausanne, Switzerland; Infectious Diseases Service, Lausanne University Hospital and University of Lausanne, Lausanne, Switzerland; Department of Infectious Diseases and Hospital Epidemiology, University Hospital Zurich and University of Zurich, Zurich, Switzerland; Department of Cardiology, Lausanne University Hospital and University of Lausanne, Lausanne, Switzerland; Department of Cardiology, Lausanne University Hospital and University of Lausanne, Lausanne, Switzerland; Department of Cardiology, Lausanne University Hospital and University of Lausanne, Lausanne, Switzerland; Department of Cardiology, University Hospital Zurich and University of Zurich, Zurich, Switzerland; Infectious Diseases Service, Lausanne University Hospital and University of Lausanne, Lausanne, Switzerland; Infection Prevention and Control Unit, Lausanne University Hospital and University of Lausanne, Lausanne, Switzerland; Department of Cardiac Surgery, Lausanne University Hospital and University of Lausanne, Lausanne, Switzerland; Department of Cardiac Surgery, Lausanne University Hospital and University of Lausanne, Lausanne, Switzerland; Department of Cardiac Surgery, University Hospital Zurich and University of Zurich, Zurich, Switzerland; Department of Cardiac Surgery, University Hospital Zurich and University of Zurich, Zurich, Switzerland; Department of Cardiac Surgery, City Hospital of Zurich—Triemli, Zurich, Switzerland; Center for Experimental and Translational Cardiology, University of Zurich, Zurich, Switzerland; Infectious Diseases Service, Lausanne University Hospital and University of Lausanne, Lausanne, Switzerland; Department of Infectious Diseases and Hospital Epidemiology, University Hospital Zurich and University of Zurich, Zurich, Switzerland; Infectious Diseases Service, Hospital of Valais and Institut Central des Hôpitaux, Sion, Switzerland

**Keywords:** antimicrobial treatment, bloodstream infection, empiric treatment, infective endocarditis, *Staphylococcus aureus*

## Abstract

**Background:**

In patients with suspected infective endocarditis (IE), current guidelines recommend prompt initiation of empiric antimicrobial treatment after obtaining blood cultures. However, the clinical benefit of immediate treatment in hemodynamically stable patients remains uncertain. This study assessed the impact of deferring antimicrobial treatment in patients with suspected IE.

**Methods:**

We conducted a multicenter cohort study of adult patients with bacteremia and clinical suspicion of IE from 2 university hospitals (2015–24). Patients presenting with sepsis, intensive care unit admission, neutropenia, or a clearly identifiably focus other than IE were excluded. All cases were adjudicated by a dedicated Endocarditis Team as either IE or not IE. The primary outcome for all episodes was 30-day mortality; for confirmed IE cases, the composite outcome included 30-day mortality, new embolic events, or new bone and joint infection.

**Results:**

Among 1230 episodes, empirical antimicrobial treatment was initiated immediately (Group I) after blood culture collection in 675 episodes (55%) and deferred until preliminary blood culture results (Group D) in 555 episodes (45%). Thirty-day mortality was 5% (59 episodes), with no difference between Groups I and D (5% vs 5%; *P* = .894). Of 597 confirmed IE episodes (49%) IE, 327 (55%) were in Group I and 270 (45%) in Group D. The composite primary endpoint occurred in 157 episodes (26%), with no difference between groups (28% vs 24%; *P* = .304).

**Conclusions:**

In clinically stable patients with suspected IE, deferring antimicrobial treatment until available blood culture results was not associated with worse clinical outcomes.

## Teaser

In this retrospective cohort study of 1230 hemodynamically stable adults with suspected infective endocarditis, deferring antimicrobial treatment until blood culture results were available was not associated with 30-day mortality. The same was observed in 597 confirmed infective endocarditis episodes.

## Graphical Abstract

**Graphical Abstract:**
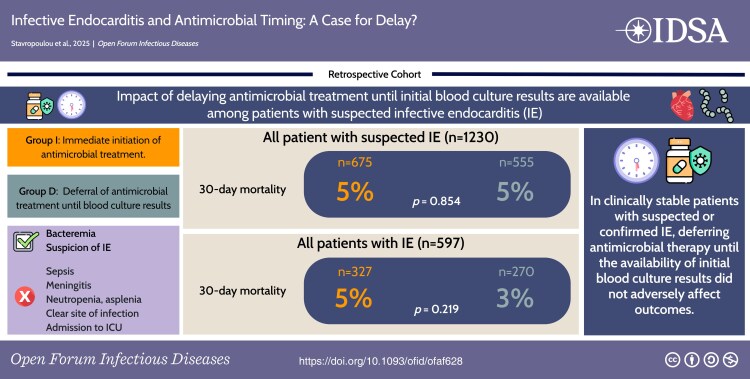
This graphical abstract is also available at Tidbit: https://tidbitapp.io/tidbits/infective-endocarditis-and-antimicrobial-timing-a-case-for-delay?utm_campaign=tidbitlinkshare&utm_source=IO

Infective endocarditis (IE) is associated with high morbidity and mortality [1, 2]. European and American guidelines recommend promptly initiating empirical antimicrobial treatment (AT) in all patients with suspected IE after obtaining 3 sets of blood cultures (BC), regardless of the severity of infection [3, 4]. This recommendation is largely based on studies from other clinical contexts showing an association between early appropriate AT and improved survival [5]. Indeed, delaying AT beyond 4–8 hours in lower respiratory tract, urinary tract, or abdominal infections delays has been linked to poorer outcomes [6]. However, these conclusions are primarily drawn from retrospective studies, whereas prospective studies have not consistently confirmed this association [6]. Furthermore, the impact of delayed AT appears to be influenced by disease severity and is typically driven by patients presenting with sepsis or septic shock [5, 7, 8].

A key concern with immediate AT in suspected IE is the high misdiagnosis rate; only 1 in 4 patients initially suspected of having IE is ultimately diagnosed with the disease, while the others are ruled out, and some of them have no infectious etiology at all [9]. Moreover, empirical AT regimens tend to be broader than targeted therapies, thereby increasing the risk of antimicrobial resistance [6]. Although the timely administration of appropriate AT is well-established as critical in cases of bacteremia or suspected IE, the evidence supporting this approach in patients with mild to moderate illnesses is limited [1, 5, 8, 10–15].

The Working Party of the British Society for Antimicrobial Chemotherapy recommends withholding antimicrobial AT in clinically stable patients with suspected IE until BC results become available [16].

Given the lack of evidence demonstrating improved outcomes from early empirical AT in clinically stable patients, this study aims to evaluate the impact of delaying AT on survival among patients with suspected and confirmed IE.

## MATERIALS AND METHODS

### Study Design and Setting

This retrospective was study conducted between January 2015 and June 2024 at 2 Swiss tertiary care centers: Lausanne University Hospital (CHUV) and University Hospital Zurich (USZ). It included patients from 3 distinct cohorts:

- CHUV retrospective bacteremia cohort (January 2015–December 2021)
- CHUV prospective cohort of patients with suspected IE (January 2022–June 2024)
- USZ endocarditis cohort (January 2015–June 2024), including retrospectively enrolled patients from 2015–2017 and prospectively enrolled patients from 2018 onward).

Suspicion of IE for all cohorts was defined as the combination of BC collection and echocardiography performed specifically to investigate IE. The study was approved by the relevant Swiss ethics committees (CER-VD 2021-02516, CER-VD 2017-02137, KEK-2014-0461; BASEC-2017-01140).

### Participants

Inclusion criteria were adult patients (≥18 years old), confirmed bacteremia, clinical suspicion of IE, and no documented objection to the use of their data. Exclusion criteria included: presence of sepsis, meningitis, neutropenia, asplenia, heart failure requiring valve surgery, clearly identifiable infectious focus other than IE, ongoing AT or unknown timing of AT initiation in relation to BC collection, or admission to an intensive care unit within 24 hours of bacteremia.

Data on demographics, clinical characteristics, imaging, microbiology, laboratory values, surgical interventions, and pathology were manually retrieved from patient's electronic health records. All data were reviewed by infectious diseases (ID) consultants. In both institutions, ID consultation was mandatory for patients with suspected IE, and follow-up BC were routinely obtained until bloodstream sterilization was confirmed. Since January 2018, the diagnosis of IE has been established by the respective institutional Endocarditis Teams (ET). For cases prior to 2018, IE adjudication was performed by 2 experienced clinicians from each center (CHUV: M. P. O., P. M.; USZ: B. H., M. F.), all of whom have been members of their institution's ET since January 2018. The site of infection other than IE was determined by the ID consultant based on an integrated assessment of clinical presentation, imaging, microbiological data, and surgical findings.

### Variables and Definitions

The date of collection of the first positive BC was defined as the onset of infection. A new episode was included if more than 30 days had passed since the completion of AT for the initial bacteremia. Classification of bacteremia cases as community-acquired, healthcare-associated, or nosocomial was based on the criteria established by Friedman et al [17]. Episodes were stratified into 2 groups: *Group I* included episodes who received empiric AT immediately after BC collection, whereas *Group D* comprised those in whom AT was deferred until preliminary BC results were available, defined as species identification by matrix-assisted laser desorption/ionization time-of-flight (MALDI-TOF) or, if inconclusive, the Gram stain result. Appropriate treatment was defined as the administration of at least 1 antimicrobial agent to which the isolated pathogens were susceptible in vitro.

Narrow-spectrum AT were defined as follows: flucloxacillin or cefazolin for monomicrobial methicillin-susceptible *Staphylococcus aureus* (MSSA) bacteremia; penicillin or amoxicillin for monomicrobial penicillin-susceptible streptococcal bacteremia; and amoxicillin for monomicrobial amoxicillin-susceptible *Enterococcus faecalis* bacteremia. The time from AT initiation to narrow-spectrum therapy was defined as the duration of broad-spectrum use.

Major embolic events included peripheral arterial embolism, septic pulmonary emboli, cerebral, ocular hepatic, renal or splenic emboli, mycotic aneurysm, Janeway lesions or nail bed hemorrhage. New bone and joint infections (BJI) included septic arthritis, acute osteomyelitis (vertebral or non-vertebral), or orthopedic implant-associated infections, with symptom onset occurring at least 24 hours after first blood culture. The date of the new BJI was defined as the date of symptom specific to that infection. Persistent bacteremia was defined as the presence of at least 1 positive BC for the same pathogen at 48 hours or more after the initial culture.

### Statistical Methods

Statistical analyses included the use of *chi*-square or Fisher's exact test for categorical variables and the Mann–Whitney *U* test for continuous variables, as appropriate. Kaplan–Meier survival curves and log-rank tests were used to evaluate 30-day mortality in all suspected or confirmed IE episodes, and the 30-day composite primary outcome (mortality, new embolic event, or new bone/joint infection) in patients with suspected or confirmed IE.

To further assess the association between AT timing and 30-day mortality, we performed univariable and multivariable Cox proportional hazards regression analyses in patients with suspected and proven IE, respectively. We also evaluated the association between AT timing and the composite primary outcome among patients with proven IE. Models were adjusted for repeated episodes within individual patients using robust standard errors. Interaction terms and potential effect modifiers were tested to explore whether the association between AT timing and outcomes differed across clinically relevant subgroups. In a sensitivity analysis, we applied inverse probability weights (IPW) for receiving immediate versus delayed AT based on logistic regression analyses using information available at admission (sex, age, comorbidities, Charlson Comorbidity Index, institution, setting of bacteremia onset, fever, temperature, embolic events at bacteremia onset, white blood cells and C-reactive protein). We conducted data analyses using Stata 19.0 SE (StataCorp, College Station, TX, USA).

## RESULTS

**Figure 1.**
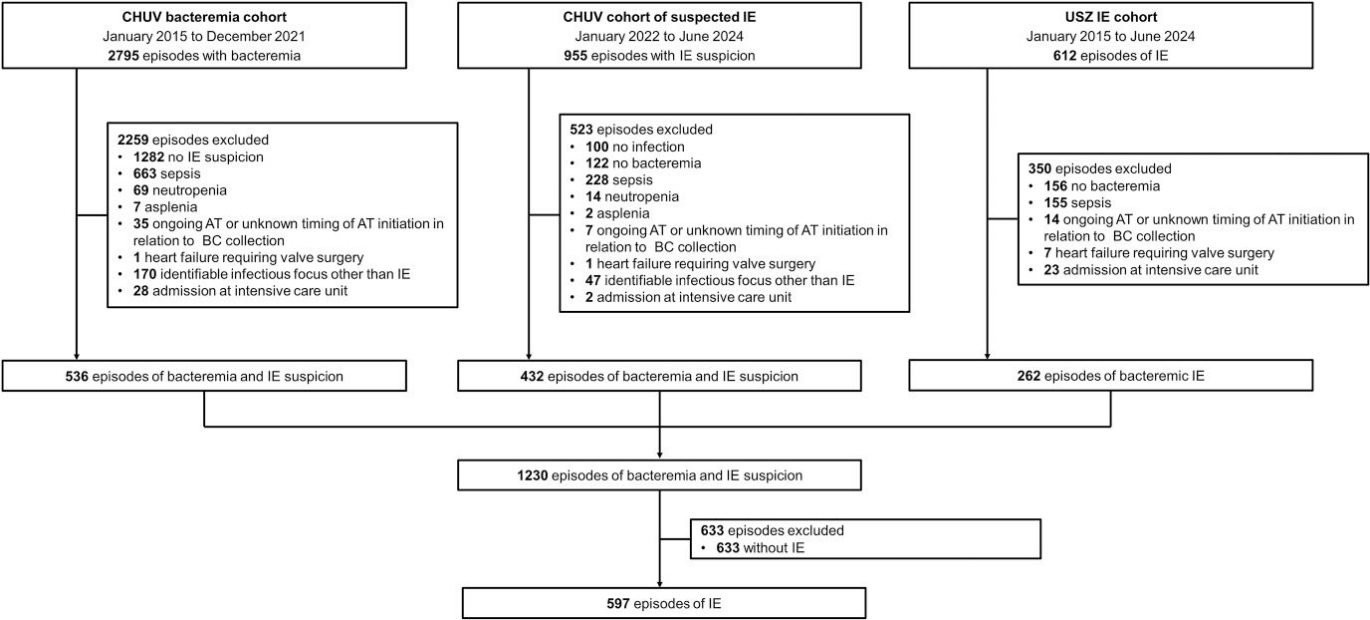
Flowchart of included episodes. Abbreviations: AT, antimicrobial treatment; BC, blood culture; CHUV, Lausanne University Hospital; IE, infective endocarditis; USZ, University Hospital Zurich. Suspicion of IE was defined as the combination of blood culture collection and echocardiography performed specifically to investigate IE.

Among the 4362 episodes, 2702 involved bacteremia and suspected IE (Figure 1). Of these, 1230 of episodes met the inclusion criteria, corresponding to 1152 individual patients (Figure 1). *S. aureus* was the most frequently isolated pathogen, identified 500 episodes (41%), followed by streptococci (318 episodes; 26%) and enterococci (203; 17%).

### Baseline Characteristics Among 1230 Episodes of Suspected IE

Transthoracic echocardiography, transesophageal echocardiography, ^18^F-FDG PET/CT, and cardiac CT were performed in 1164 (95%), 677 (55%), 215 (18%), and 39 (3%) episodes, respectively. The timing from blood culture collection to the first echocardiography was similar between the 2 centers (CHUV: median 2 days, interquartile range 1–3; USZ: median 2 days, interquartile range 1–4). Overall, IE was diagnosed in 597 episodes (49%) by the ETs or expert clinicians. Of these, IE involved native valves in 400 (67%), prosthetic valves in 156 (26%), and cardiac implantable electronic device leads in 71 (12%) episodes. Among the 633 episodes not diagnosed with IE, the most common alternative diagnoses were catheter-related bloodstream infection (219 episodes; 35%), BJI (190; 30%), and bacteremia of unknown origin (105; 17%).

Immediate AT (*Group I*) was initiated in 675 (55%) episodes, whereas AT was deferred until preliminary BC results became available (*Group D*) in 555 (45%) episodes (Table 1). In *Group D*, AT was initiated within 24 hours in 405 (73%) episodes, within 24–48 hours in 110 (20%) episodes, and within 48–72 hours in 40 (7%) episodes. Among the latter, time to BC positivity data were available in 20 cases; all of which had a time to positivity of ≥36 hours. A higher proportion of episodes in *Group I* received appropriate AT within 48 hours of BC collection compared to *Group D* [653 (97%) of 675 episodes *versus* 501 (90%) of 555 episodes; *P* < .001]. Source control interventions were deemed necessary in 580 episodes (47%) and were successfully performed in 299 of these cases (52%). The rate of source control intervention, when indicated, was similar between *Groups I* and *D* [170 (52%) of 329 episodes *versus* 129 (51%) of 251 episodes; *P* = 1.000].

**Table 1.** Comparison Based on the Timing of Antimicrobial Treatment Initiation Among Episodes With Suspected Infective Endocarditis

	Group D (n = 555)	Group I (n = 675)	*P*
Demographics			
Male sex, n (%)	399 (72)	481 (71)	.849
Age (years), median (IQR)	65 (53–76)	68 (52–78)	.101
Age >60 y, n (%)	350 (63)	440 (65)	.473
Co-morbidities			
Diabetes mellitus, n (%)	122 (22)	158 (23)	.585
Obesity (body mass index ≥30 kg/m^2^), n (%)	99 (18)	137 (20)	.308
Chronic kidney disease (moderate or severe), n (%)	130 (23)	173 (26)	.388
Malignancy (solid organ or hematologic), n (%)	98 (18)	111 (16)	.594
Immunosuppression, n (%)^a^	67 (12)	83 (12)	.930
Chronic obstructive pulmonary disease, n (%)	67 (12)	68 (10)	.273
Cirrhosis, n (%)	65 (12)	68 (10)	.358
Congestive heart failure, n (%)	79 (14)	70 (10)	.043
Intravenous drug use, n (%)	53 (10)	42 (6)	.032
Prior infective endocarditis, n (%)	42 (8)	45 (7)	.577
Surgical prosthetic valve or TAVI, n (%)	95 (17)	119 (18)	.821
Cardiac implantable electronic device, n (%)	72 (13)	93 (14)	.737
Charlson Comorbidity Index (points), median (IQR)	4 (1–6)	4 (1–6)	.101
Charlson Comorbidity Index >4 points, n (%)	238 (43)	305 (45)	.420
Hospital^b^			.001
Lausanne University Hospital, n (%)	412 (74)	555 (82)	
University Hospital Zurich, n (%)	143 (26)	120 (18)	
Setting of bacteremia onset			.036
Community, n (%)	316 (57)	425 (63)	
Healthcare-associated, n (%)	78 (14)	97 (14)	
Nosocomial, n (%)	161 (29)	153 (23)	
Microbiological data			
Three or more positive blood culture sets, n (%)	83 (15)	116 (17)	.312
Pathogens			
*S. aureus*, n (%)	205 (37)	295 (44)	.017
Coagulase-negative staphylococci, n (%)	65 (12)	52 (8)	.019
Streptococci, n (%)	132 (24)	186 (28)	.150
Enterococci, n (%)	115 (21)	88 (13)	<.001
Other Gram-positive, n (%)	18 (3)	28 (4)	.452
HACEK, n (%)	8 (1)	17 (3)	.225
Gram-negative other than HACEK, n (%)	36 (7)	47 (7)	.820
Polymicrobial bacteremia, n (%)	37 (7)	48 (7)	.822
Infection data at bacteremia onset			
Fever, n (%)	436 (79)	548 (81)	.725
Temperature (°C), median (IQR)^c^	38.3 (37.4–38.9)	38.5 (37.8–39.0)	.001
Embolic events, n (%)	86 (16)	111 (16)	.696
Cerebral embolic events, n (%)	42 (8)	47 (7)	.740
Laboratory values at bacteremia onset			
White blood cells (×10^9^/L), median (IQR)^d^	10.2 (7.2–13.2)	10.5 (7.7–14.0)	.081
C-reactive protein (mg/l), median (IQR)^e^	93 (48–170)	115 (58–213)	.001
Focus of infection			
Infective endocarditis, n (%)	270 (49)	327 (48)	.954
Catheter-related, n (%)	114 (21)	117 (17)	.164
Bone and joint infection, n (%)	110 (20)	168 (25)	.040
Other focus, n (%)	95 (17)	81 (12)	.011
Unknown focus, n (%)	37 (7)	68 (10)	.040
Management			
Source control			.469
Not warranted, n (%)	304 (55)	346 (51)	
Warranted and performed within 48 h, n (%)	129 (23)	170 (25)	
Warranted and not performed within 48 h, n (%)	122 (22)	159 (24)	
Antimicrobial treatment			
Appropriate antimicrobial treatment within 48 h, n (%)	501 (90)	653 (97)	<.001
Timing of antimicrobial treatment initiation			<.001
Within 24 h, n (%)	405 (73)	675 (100)	
Within 24–48 h, n (%)	110 (20)	0 (0)	
Within 48–72 h, n (%)	40 (7)	0 (0)	
Outcomes			
Persistent bacteremia for at least 48 h from first positive blood culture, n (%)	156 (28)	147 (22)	.011
Persistent bacteremia for at least 48 h from antimicrobial treatment onset, n (%)	106 (19)	147 (22)	.257
Bone and joint infection within 30 d, n (%)	7 (1)	11 (2)	.641
Death within 15 d, n (%)	9 (2)	15 (2)	.537
Death within 30 d, n (%)	26 (5)	33 (5)	.894
Death within 90 d, n (%)	64 (12)	65 (101)	.322

Abbreviations: IQR, interquartile range; Group I, empiric antimicrobial treatment was initiated immediately after the blood culture collection; Group D, antimicrobial treatment was deferred until the first result of blood cultures; TAVI, transcatheter aortic valve implantation.

^a^Ongoing immunosuppressive treatment at bacteremia onset, intravenous chemotherapy in the 30 d prior to bacteremia onset, or AIDS.

^b^In the 2 cohorts from Lausanne University Hospital, episodes with suspected infective endocarditis were included, whereas the cohort from University Hospital Zurich included only patients with a confirmed diagnosis of infective endocarditis.

^c^Temperature available in 1201/1230 (98%) episodes.

^d^White blood cells available in 1171/1230 (95%) episodes.

^e^C-reactive protein available in 1117/1230 (91%) episodes.

Supplementary Table 1 presents data on the initiation of narrow-spectrum AT in cases of monomicrobial bacteremia caused by MSSA, penicillin-susceptible streptococci, or amoxicillin-susceptible *E. faecalis*. Among 863 episodes, a comparable proportion of episodes in *Group I* and *Group D* received narrow-spectrum treatment [398/476 (84%) versus 328/378 (87%); *P* = .061]. However, narrow-spectrum AT was initiated later in *Group I* (mean 2.8 ± 5.7 days) than in *Group D* (mean 1.7 ± 3.4; *P* < .001). Consequently, the duration of broad-spectrum AT was longer in *Group I* (mean 2.8 ± 5.7) compared to *Group D* (mean 1.5 ± 3.4; *P* < .001).

### Baseline Characteristics Among 597 IE Episodes

Of the 1137 episodes of proven bacteremic IE, 597 (53%) met the inclusion criteria. Immediate AT (*Group I*) was initiated in 327 (55%) episodes, and deferred (*Group D*) in 270 (45%) episodes (Supplementary Table 2). Supplementary Table 3 presents data on the initiation of narrow-spectrum AT in 451 episodes of monomicrobial IE caused by MSSA, penicillin-susceptible streptococci, or amoxicillin-susceptible *E. faecalis*. A smaller proportion of episodes in *Group I* received narrow-spectrum agents compared to *Group D* [193/250 (77%) versus 172/201 (86%); *P* = .030]. Narrow-spectrum AT was initiated later in *Group I* (mean 3.2 ± 7.1 days) than in *Group D* (2.2 ± 4.2 days; *P* = .067), and the duration of broad-spectrum AT was longer in *Group I* (3.2 ± 7.1 days) compared to *Group D* (1.9 ± 4.2 days; *P* = .001).

### Outcome Among 1230 Episodes With Suspected IE

**Figure 2.**
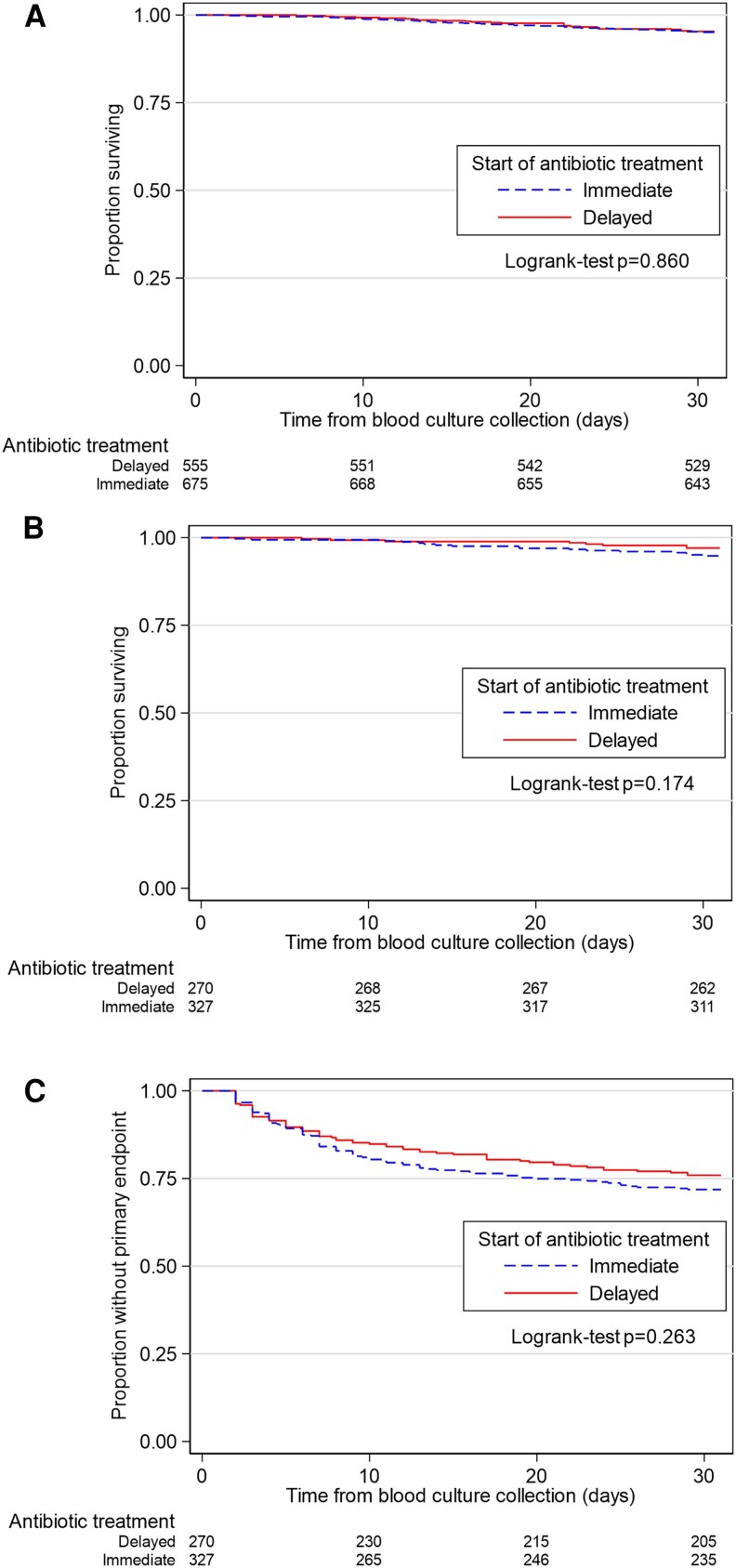
Kaplan–Meier analysis based on the timing of antimicrobial treatment initiation: *A*, 30-day mortality among all 1230 episodes (log-rank test: *P* = .854); *B*, 30-day mortality among 597 infective endocarditis episodes (log-rank test: *P* = .174); and *C*, 30-day primary endpoint (composite of mortality, new embolic event, or new bone and joint infection) among 597 infective endocarditis episodes (log-rank test: *P* = .263).

Thirty-day mortality was 5% (59 episodes). Kaplan–Meier analysis showed no evidence of a difference in 30-day mortality between *Group I* and *Group D* [26 (5%) out of 675 episodes *versus* 33 (5%) out of 555 episodes; log-rank test *P* = .854] (Figure 2*A*).

**Figure 3.**
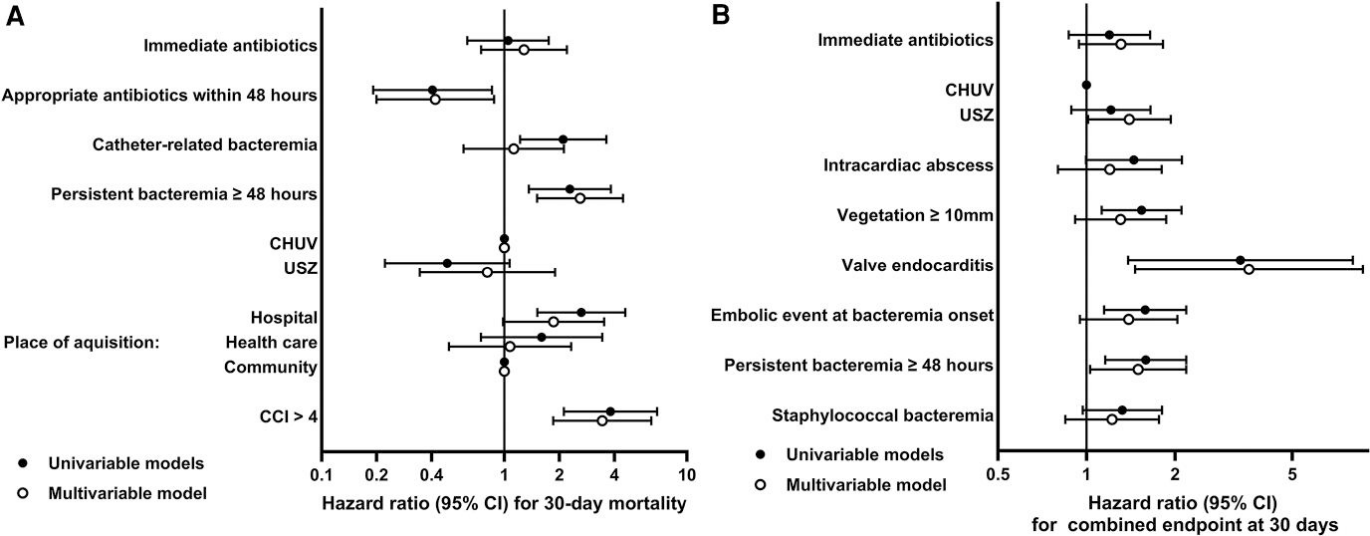
Uni- and multivariable Cox regression analysis of *A*, 30-day mortality among all 1230 episodes, and *B*, 30-day primary endpoint (composite of mortality, new embolic event, or new bone and joint infection) among 597 infective endocarditis episodes. Abbreviations: CHUV, Lausanne University Hospital; USZ, University Hospital Zurich.

Supplementary Table 4 compares baseline characteristics between 30-day survivors and non-survivors among episodes with suspected IE. Figure 3*A* and Supplementary Table 5 show the uni- and multivariable Cox regression analysis of 30-day mortality among the same patients. In the multivariable model, independent predictors of increased 30-day mortality included Charlson Comorbidity Index >4 (aHR 3.43; 95% CI 1.85–6.36) and persistent bacteremia (2.60; 1.51–4.46). Appropriate treatment within 48 hours remained protective (0.42; 0.20–0.88). Immediate initiation of antimicrobial treatment was not associated with lower 30-day mortality (1.28; 0.75–2.20). In the sensitivity analysis of 1081 episodes with suspected IE and with complete baseline information, the logistic regression to calculate the probability of initiating immediate AT yielded a poor to moderate model performance with a ROC AUC of 0.65. The Cox regression analysis with IPW showed that immediate AT was not associated with lower 30-day mortality (0.99; 0.56–1.74).

### Outcome Among 597 Episodes With IE

The composite 30-day primary endpoint occurred in 157 episodes (26%). Specifically, 30-day mortality was 4% (25 episodes), new embolic events occurred in 134 episodes (22%), and new BJIs in 11 episodes (2%). Kaplan–Meier analyses showed no evidence of a difference regarding 30-day mortality (log-rank test: *P* = .174) (Figure 2*B*), or primary endpoint (log-rank test: *P* = .263) (Figure 2*C*).

Supplementary Table 6 compares baseline characteristics between episodes that did and did not meet the 30-day composite primary endpoint among IE episodes. Multivariable analysis identified as independent predictors of the composite primary outcome persistent bacteremia (aHR 1.50; 95% CI 1.03–2.18), USZ (1.39; 1.01–1.93), and valve endocarditis (3.56; 1.46–8.68) (Figure 3*B* and Supplementary Table 7). Supplementary Table 8 show the uni- and multivariable Cox regression analysis of 30-day mortality among IE episodes. Immediate initiation of antimicrobial treatment was not associated with lower composite primary endpoint (1.32; 0.95–1.83) nor with lower 30-day mortality (2.51; 0.93–6.78). In the 505 IE episodes with complete baseline information, the IPW Cox regression analysis (ROC AUC 0.7) showed that immediate AT was neither associated with a lower composite primary endpoint (1.10; 0.76–1.61) nor with lower 30-day mortality (0.81; 0.38–2.41).

## DISCUSSION

In this large, combined cohort of clinically stable patients with suspected IE, we found no evidence that deferring AT was associated with worse outcomes compared to immediate AT. These findings were consistent even when focusing specifically on clinically stable patients with confirmed IE, who comprised approximately half of the study population.

The decision to initiate AT appeared driven by clinical presentation rather than age or comorbidities, as baseline characteristics were comparable between groups. Immediate AT was more frequently observed in patients with *S. aureus* bacteremia, consistent with its acute presentation, while *Enterococcus* spp., generally causing a more indolent course, was associated with delayed AT [18, 19].

In *Group D*, 73% received AT within 24 hours, and 90% within 48 hours, compared to 97% in *Group I*. Delays beyond 48 hours were mainly due to late BC positivity and the time required for species identification. Notably, in most cases of monomicrobial bacteremia, narrow-spectrum AT was initiated within 2 days of BC collection, facilitated by rapid diagnostics [20–22].

In our analysis, timing of AT administration was not associated with 30-day mortality. These findings challenge current European and American guidelines [3, 4], which recommend immediate AT in all patients with suspected IE, regardless of disease severity. Mortality instead correlated with higher Charlson Comorbidity Index, persistent bacteremia beyond 48 hours, and inappropriate AT within the first 48 hours [1, 5, 8, 10–15]. Source control procedures rates, critical for bacteremia clearance and recognized predictor of clinical outcomes, were similar across groups [13–15, 23]. Although persistent bacteremia at 48 hours was more common in *Group D*, this association disappeared after adjusting for AT. Prior studies highlighted the importance of appropriate AT in bacteremia, particularly among severely ill patients [5, 8, 10, 11, 13–15]. However, in patients with less severe presentation, limited to under 24 hours have not been linked to increased mortality [8, 10, 11]. Our findings align with this evidence, showing that short delays in stable patients with suspected IE did not worsen outcomes. A similar observation was seen in *S. aureus* bacteremia, where delayed treatment was associated with poorer outcomes, only in patients with severe presentation [24].

One key advantages of withholding empiric AT in stable patients is the support of antimicrobial stewardship efforts [6]. A major rationale for deferring AT in stable patients also lies in the relatively low diagnostic yield of the Duke criteria. Nearly half of patients were ultimately diagnosed with a condition other than IE. Similarly, a previous study involving 3127 patients with suspected IE found that only 38% were ultimately diagnosed with IE. In contrast, 43% had an alternative bacterial infection, and 19% had a non-infectious condition [9]. Many of the alternative bacterial infections identified did not require immediate AT, and patients with non-infectious diagnoses will not benefit from AT. Immediate AT in all suspected IE cases would result in substantial unnecessary antimicrobial exposure. In contrast, withholding treatment allowed for more targeted and judicious use of AT, as evidenced by the earlier and more frequent use of narrow-spectrum agents among patients in *Group D*.

Beyond mortality, a major concern in IE is the risk of new embolic events, though incidence typically declines after the first 2 weeks of treatment [25, 26]. In this study, no difference was observed in new embolic events between *Groups I* and *D*. Both groups had comparable proportions of embolic events at the time of BC collection and similar rates of large vegetations, which are strong predictors of embolic complications during treatment [1, 25–27]. These findings suggest that deferring AT until BC results does not increase the risk of new embolic events or secondary BJI.

This study has limitations. The study conducted within a single healthcare system, where patients had access to consistent ID consultation and specialized ETs. Both institutions used MALDI-TOF technology directly from positive BCs, thus reducing the time to species identification [21]. These factors may limit the generalizability of our findings to other healthcare settings. Selection bias is inherent, as the decision to initiate or defer AT was based on complex clinical judgments not fully captured in the available data. Although we adjusted for numerous baseline variables using IPW, residual confounding by unmeasured factors cannot be excluded. Nonetheless, factors such as regional differences in clinical practice and antimicrobial stewardship policies may have influenced our results. Patients at CHUV were more likely to receive immediate AT, whereas at USZ, deferral of treatment until BC results was more common, consistent with previously reported regional variations in antimicrobial use in Switzerland [28, 29]. Moreover, in the majority of episodes, suspicion of IE did not arise at initial presentation, but only after the first blood culture results were available, as previously shown in a Swedish study that found that IE was suspected at presentation in only 23% of cases [30]. However, the exact timing of when IE was first suspected, as well as the interval between patient presentation and the collection of the initial blood cultures, was not systematically recorded. Safety outcomes, including adverse drug events such as *Clostridioides difficile* infection, were not systematically recorded, limiting comparisons of treatment-related harms between groups. Lastly, parameters reflecting medical decisions, such as the immediate initiation of antimicrobial treatment, are based on complex clinical evaluations that are not fully captured during data collection.

While early empirical AT may accelerate microbiological clearance, our findings suggest that deferring AT until initial BC results in clinically stable patients with suspected or confirmed IE did not negatively affect mortality or embolic complications. These findings advocate for a careful, patient-centered approach to initiating AT in clinically stable patients with suspected IE, particularly in cases with diagnostic uncertainty helping to avoid unnecessary use of broad-spectrum antimicrobials. Importantly, immediate AT remains essential for patients with sepsis, neutropenia, asplenia, or a clear alternative infectious focus. In contrast, the mere suspicion of IE in otherwise stable patients should not automatically trigger AT. These findings underscore the potential for antimicrobial stewardship even in high-stakes scenarios, but should be validated in external cohorts, especially in settings with a high burden of antimicrobial resistance.

## Supplementary Material

ofaf628_Supplementary_Data

## Supplementary Data

Supplementary materials are available at *Open Forum Infectious Diseases* online. Consisting of data provided by the authors to benefit the reader, the posted materials are not copyedited and are the sole responsibility of the authors, so questions or comments should be addressed to the corresponding author.
