# Supplementary material for: Infective Endocarditis and Antimicrobial Timing: A Case for Delay?
Source: Open Forum Infect Dis. 2025 Oct 7;12(10):ofaf628. doi: 10.1093/ofid/ofaf628 (PMC12548369; doi:10.1093/ofid/ofaf628)
Supplement: ofaf628_Supplementary_Data [file ofaf628_supplementary_data.pdf]

Supplementary Table 1. Timing of initiation of narrow spectrum antimicrobials for monomicrobial bacteremia due to methicillin-susceptible *S. aureus*, penicillin-susceptible streptococci, or amoxicillin-susceptible *E. faecalis*.

	**Group D**	**Group I**	***P***
**Monomicrobial bacteremia due to methicillin-susceptible *S. aureus***	**N=185**	**N=268**	
Administration of flucloxacillin or cefazolin, n (%)	175 (95)	257 (96)	0.650
Time of initiation of flucloxacillin or cefazolin, mean days (SD)	1.2 (1.5)	1.5 (1.6)	0.059
Days of antimicrobial treatment on broad-spectrum antibiotics, mean days (SD)	1.0 (1.3)	1.5 (1.6)	0.001
**Monomicrobial bacteremia due to penicillin-susceptible streptococci**	**N=108**	**N=159**	
Administration of penicillin or amoxicillin, n (%)	80 (74)	91 (57)	0.006
Time of initiation of penicillin or amoxicillin, mean days (SD)	2.6 (5.6)	5.8 (10.3)	0.002
Days of antimicrobial treatment on broad-spectrum antibiotics, mean days (SD)	2.2 (5.5)	5.8 (10.3)	<0.001
**Monomicrobial bacteremia due to amoxicillin-susceptible *E. faecalis***	**N=88**	**N=55**	
Administration of amoxicillin, n (%)	76 (86)	47 (86)	1.000
Time of initiation of amoxicillin, mean days (SD)	2.0 (3.6)	3.9 (5.)	<0.001
Days of antimicrobial treatment on broad-spectrum antibiotics, mean days (SD)	1.8 (3.5)	3.9 (5.)	<0.001
**All aforementioned pathogens**	**N=378**	**N=485**	
Administration of narrow spectrum antimicrobials, n (%)	328 (87)	398 (82)	0.061
Time of initiation of narrow spectrum antimicrobials, mean days (SD)	1.7 (3.4)	2.8 (5.7)	<0.001

Days of antimicrobial treatment on broad-spectrum antibiotics, mean days (SD)	1.5 (3.4)	2.8 (5.7)	<0.001

Group I: empiric antimicrobial treatment was initiated immediately after the blood culture collection; Group D: antimicrobial treatment was deferred until the first result of blood cultures; SD: standard deviation

Supplementary Table 2. Comparison based on the timing of antimicrobial treatment initiation among 597 episodes with infective endocarditis

	**Group D (n=270)**	**Group I (n=327)**	***P***
Demographics			
Male sex, n (%)	203 (75)	244 (75)	0.925
Age (years), median (IQR)	65 (47-75)	67 (51-80)	0.164
Age >60 years, n (%)	155 (57)	211 (65)	0.077
Co-morbidities			
Diabetes mellitus, n (%)	51 (19)	71 (22)	0.416
Obesity (body mass index ≥30 kg/m^2), n (%)	38 (14)	64 (20)	0.081
Chronic kidney disease (moderate or severe), n (%)	63 (23)	70 (21)	0.621
Malignancy (solid organ or hematologic), n (%)	37 (14)	40 (12)	0.625
Immunosuppression, n (%)[a]	19 (7)	21 (6)	0.870
Chronic obstructive pulmonary disease, n (%)	26 (10)	29 (9)	0.777
Cirrhosis, n (%)	42 (16)	31 (10)	0.032
Congestive heart failure, n (%)	52 (19)	35 (11)	0.004
Intravenous drug use, n (%)	28 (10)	17 (5)	0.019
Prior infective endocarditis, n (%)	33 (12)	33 (10)	0.433
Surgical prosthetic valve or TAVI, n (%)	78 (29)	95 (29)	1.000

Cardiac implantable electronic device, n (%)	49 (18)	69 (21)	0.409
Charlson Comorbidity Index (points), median (IQR)	3 (1-5)	3 (1-5)	0.587
Charlson Comorbidity Index >4 points, n (%)	88 (33)	112 (34)	0.728
Hospital			<0.001
Lausanne University Hospital, n (%)	127 (47)	210 (64)	
University Hospital Zurich, n (%)	143 (53)	117 (36)	
Setting of bacteremia onset			0.768
Community, n (%)	218 (81)	265 (81)	
Healthcare-associated, n (%)	25 (9)	34 (10)	
Nosocomial, n (%)	27 (10)	28 (9)	
Microbiological data			
Three or more positive blood culture sets, n (%)	61 (23)	85 (26)	0.341
Pathogens			
S. aureus, n (%)	72 (27)	113 (35)	0.041
Coagulase-negative staphylococci, n (%)	28 (10)	22 (7)	0.137
Streptococci, n (%)	89 (33)	113 (35)	0.728
Enterococci, n (%)	62 (23)	51 (16)	0.027
Other Gram-positive, n (%)	10 (4)	9 (3)	0.641
HACEK, n (%)	7 (3)	14 (4)	0.372

Gram-negative other than HACEK, n (%)	7 (3)	11 (3)	0.638
Polymicrobial bacteremia, n (%)	9 (3)	13 (4)	1.000
Infection data at bacteremia onset			
Fever, n (%)	219 (81)	268 (82)	0.832
Temperature (ºC), median (IQR)[b]	38.1 (37.2-38.8)	38.4 (37.6-39.0)	<0.001
Embolic events, n (%)	79 (29)	92 (28)	0.785
Cerebral embolic events, n (%)	42 (16)	45 (14)	0.561
Bone and joint infection, n (%)	23 (9)	39 (12)	0.181
Laboratory values at bacteremia onset			
White blood cells ($\times 10^9$/L), median (IQR)[c]	10.1 (7.6-12.8)	10.3 (8.2-13.2)	0.158
C-reactive protein (mg/l), median (IQR)[d]	84 (47-143)	113 (61-203)	0.001
Site of infective endocarditis			
Valve endocarditis, n (%)	247 (92)	297 (91)	0.885
Aortic valve, n (%)	133 (49)	165 (51)	0.805
Mitral valve, n (%)	109 (40)	138 (42)	0.677
Tricuspid valve, n (%)	19 (7)	33 (7)	1.000
Pulmonary valve, n (%)	8 (3)	7 (2)	0.604
Cardiac implantable electronic device -lead, n (%)	34 (13)	37 (11)	0.703
Type of valve			

Native, n (%)	182 (67)	227 (69)	0.658
Prosthetic, n (%)	68 (25)	81 (25)	0.925
Intracardiac lesions			
Vegetation, n (%)	195 (72)	218 (67)	0.155
Vegetation ≥10mm, n (%)	101 (37)	120 (37)	0.865
Abscess, n (%)	49 (18)	50 (15)	0.377
Other lesions, n (%)[c]	36 (13)	40 (12)	0.712
Management			
Source control			0.484
Not warranted, n (%)	212 (79)	243 (74)	
Warranted and performed within 48h, n (%)	10 (4)	15 (5)	
Warranted and not performed within 48h, n (%)	48 (18)	69 (21)	
Antimicrobial treatment			
Appropriate antimicrobial treatment within 48h, n (%)	244 (90)	320 (98)	<0.001
Timing of antimicrobial treatment initiation, n (%)			<0.001
Within 24h, n (%)	192 (71)	327 (100)	
Within 24-48h, n (%)	53 (20)	0 (0)	
Within 48-72h, n (%)	25 (9)	0 (0)	
Outcomes			

Primary endpoint, n (%)	65 (24)	92 (28)	0.304
Death within 30 days, n (%)	8 (3)	17 (5)	0.219
Embolic events within 30 days, n (%)	57 (21)	77 (24)	0.492
Bone and joint infection within 30 days, n (%)	5 (2)	6 (2)	1.000
Persistent bacteremia for at least 48h from first positive blood culture, n (%)	95 (35)	85 (26)	0.016
Persistent bacteremia for at least 48h from antimicrobial treatment onset, n (%)	62 (23)	85 (26)	0.445
Death within 15 days, n (%)	3 (1)	8 (2)	0.360
Death within 90 days, n (%)	20 (7)	33 (5)	0.311

[a]ongoing immunosuppressive treatment at bacteremia onset, intravenous chemotherapy in the 30 days prior to bacteremia onset, or AIDS.

[b]Temperature available in 568/597 (95%) episodes

[c]White blood cells available in 547/597 (92%) episodes

[d]C-reactive protein available in 539/597 (90%) episodes

IQR: interquartile range; Group I: empiric antimicrobial treatment was initiated immediately after the blood culture collection; Group D: antimicrobial treatment was deferred until the first result of blood cultures; TAVI: transcatheter aortic valve implantation

Supplementary Table 3. Timing of initiation of narrow spectrum antimicrobials for monomicrobial endocarditis due to methicillin-susceptible *S. aureus*, penicillin-susceptible streptococci, or amoxicillin-susceptible *E. faecalis*.

	Group W	Group I	*P*
**Monomicrobial bacteremia due to methicillin-susceptible *S. aureus***	**N=68**	**N=103**	
Administration of flucloxacillin or cefazolin, n (%)	62 (91)	94 (91)	1.000
Time of initiation of flucloxacillin or cefazolin, mean days (SD)	1.6 (1.5)	1.6 (1.5)	0.610
Days of antimicrobial treatment on broad-spectrum antibiotics, mean days (SD)	1.4 (1.3)	1.6 (1.5)	0.799
**Monomicrobial bacteremia due to penicillin-susceptible streptococci**	**N=79**	**N=103**	
Administration of penicillin or amoxicillin, n (%)	62 (79)	59 (57)	0.003
Time of initiation of penicillin or amoxicillin, mean days (SD)	2.4 (5.9)	5.2 (11.6)	0.066
Days of antimicrobial treatment on broad-spectrum antibiotics, mean days (SD)	2.1 (5.8)	5.2 (11.6)	0.005
**Monomicrobial bacteremia due to amoxicillin-susceptible *E. faecalis***	**N=54**	**N=44**	
Administration of amoxicillin, n (%)	48 (89)	40 (91)	1.000
Time of initiation of amoxicillin, mean days (SD)	2.5 (4.3)	4.1 (5.3)	0.013
Days of antimicrobial treatment on broad-spectrum antibiotics, mean days (SD)	2.2 (4.2)	4.1 (5.3)	0.002
**All aforementioned pathogens**	**N=201**	**N=250**	
Administration of narrow spectrum antimicrobials, n (%)	172 (86)	193 (77)	0.030
Time of initiation of narrow spectrum antimicrobials, mean days (SD)	2.2 (4.2)	3.2 (7.1)	0.067

Days of antimicrobial treatment on broad-spectrum antibiotics, mean days (SD)	1.9 (4.2)	3.2 (7.1)	0.001

Group I: empiric antimicrobial treatment was initiated immediately after the blood culture collection; Group D: antimicrobial treatment was deferred until the first result of blood cultures; SD: standard deviation

Supplementary Table 4. Comparison of 30-day survivors and non-survivors among 1230 episodes with suspected infective endocarditis

	**Survivors (n=1171)**	**Non-survivors (n=59)**	***P***
Demographics			
Male sex, n (%)	840 (72)	40 (68)	0.554
Age (years), median (IQR)	66 (51-77)	74 (68-83)	<0.001
Age >60 years, n (%)	738 (63)	52 (88)	<0.001
Co-morbidities			
Diabetes mellitus, n (%)	260 (22)	20 (34)	0.054
Obesity (body mass index ≥30 kg/m^2), n (%)	222 (19)	14 (23)	0.396
Chronic kidney disease (moderate or severe), n (%)	281 (24)	22 (37)	0.029
Malignancy (solid organ or hematologic), n (%)	193 (17)	16 (27)	0.048
Immunosuppression, n (%)[a]	137 (12)	13 (22)	0.024
Chronic obstructive pulmonary disease, n (%)	128 (11)	7 (12)	0.830
Cirrhosis, n (%)	125 (11)	8 (14)	0.516
Congestive heart failure, n (%)	141 (12)	8 (14)	0.684
Intravenous drug use, n (%)	94 (8)	1 (2)	0.081
Prior infective endocarditis, n (%)	84 (7)	3 (5)	0.794
Surgical prosthetic valve or TAVI, n (%)	202 (17)	12 (20)	0.597

Cardiac implantable electronic device, n (%)	157 (13)	8 (14)	1.000
Charlson Comorbidity Index (points), median (IQR)	4 (1-6)	6 (5-8)	<0.001
Charlson Comorbidity Index >4 points, n (%)	499 (43)	44 (75)	<0.001
Hospital[b]			0.074
Lausanne University Hospital, n (%)	915 (78)	52 (88)	
University Hospital Zurich, n (%)	256 (22)	7 (12)	
Setting of bacteremia onset			0.002
Community, n (%)	717 (62)	24 (41)	
Healthcare-associated, n (%)	166 (14)	9 (15)	
Nosocomial, n (%)	288 (25)	26 (44)	
Microbiological data			
Three or more positive blood culture sets, n (%)	194 (16)	7 (12)	0.468
Pathogens			
S. aureus, n (%)	476 (41)	24 (41)	1.000
Coagulase-negative staphylococci, n (%)	107 (9)	10 (17)	0.064
Streptococci, n (%)	308 (26)	10 (17)	0.128
Enterococci, n (%)	190 (16)	13 (22)	0.279
Other Gram-positive, n (%)	43 (4)	3 (5)	0.481
HACEK, n (%)	24 (2)	1 (2)	1.000

Gram-negative other than HACEK, n (%)	77 (8)	6 (10)	0.282
Polymicrobial bacteremia, n (%)	77 (7)	8 (14)	0.059
Persistent bacteremia for at least 48h from first positive blood culture, n (%)	278 (24)	25 (42)	0.003
Infection data at bacteremia onset			
Fever, n (%)	942 (80)	42 (71)	0.095
Embolic events, n (%)	185 (16)	12 (20)	0.363
Cerebral embolic events, n (%)	84 (7)	5 (9)	0.610
Focus of infection			
Infective endocarditis, n (%)	572 (49)	25 (42)	0.353
Catheter-related, n (%)	212 (18)	19 (32)	0.010
Bone and joint infection, n (%)	271 (23)	7 (12)	0.054
Other focus, n (%)	166 (14)	10 (17)	0.567
Unknown focus, n (%)	100 (9)	5 (9)	1.000
Management			
Source control			0.114
Not warranted, n (%)	624 (53)	26 (44)	
Warranted and performed within 48h, n (%)	286 (24)	13 (22)	
Warranted and not performed within 48h, n (%)	261 (22)	20 (34)	
Antimicrobial treatment			

Timing of antimicrobial treatment initiation			0.894
Group I: immediate initiation, n (%)	642 (55)	33 (56)	
Group D: deferring after blood culture result, n (%)	549 (45)	26 (894)	
Appropriate antimicrobial treatment within 48h, n (%)	1103 (94)	51 (86)	0.025

[a]ongoing immunosuppressive treatment at bacteremia onset, intravenous chemotherapy in the 30 days prior to bacteremia onset, or AIDS.

[b]In the two cohorts from Lausanne University Hospital, episodes with suspected infective endocarditis were included, whereas the cohort from University Hospital Zurich included only patients with a confirmed diagnosis of infective endocarditis

IQR: interquartile range; Group I: empiric antimicrobial treatment was initiated immediately after the blood culture collection; Group D: antimicrobial treatment was deferred until the first result of blood cultures; TAVI: transcatheter aortic valve implantation

Supplementary Table 5. Univariable and multivariable Cox proportional hazard regression of 30-day mortality among 1230 episodes with suspected infected endocarditis

	Univariable analysis		Multivariable Cox regression	
	P	**HR (95% CI)**	***P***	**aHR (95% CI)**
Charlson Comorbidity Index >4 points	<0.001	3.81 (2.12-6.85)	<0.001	3.43 (1.85-6.36)
Hospital				
Lausanne University Hospital	reference		reference	
University Hospital Zurich	0.072	0.49 (0.22-1.07)	0.623	0.81 (0.34-1.89)
Setting of bacteremia onset				
Community	reference		reference	
Healthcare-associated	0.229	1.60 (0.74-3.43)	0.855	1.07 (0.50-2.32)
Nosocomial	0.001	2.64 (1.51-4.59)	0.057	1.86 (0.98-3.51)
Persistent bacteremia for ≥48 hours from bacteremia onset	0.002	2.28 1.36-3.83)	0.001	2.60 (1.51-4.46)
Catheter related bacteremia	0.008	2.10 (1.22-3.62)	0.716	1.12 (0.60-2.11)
Appropriate antimicrobial treatment within 48 hours	0.018	0.40 (0.19-0.86)	0.021	0.42 (0.20-0.88)
Immediate initiation of antimicrobial treatment	0.859	1.05 (0.63-1.75)	0.368	1.28 (0.75-2.20)

CI: confidence interval; aHR: adjusted hazard ratio

Supplementary Table 6. Comparison of episodes that did and did not meet the 30-day primary endpoint (composite of mortality, new embolic event, new bone and joint infection) among 597 episodes with infective endocarditis

	**No primary outcome (n=440)**	**Primary outcome (n=157)**	***P***
Demographics			
Male sex, n (%)	327 (74)	120 (76)	0.668
Age (years), median (IQR)	65 (49-76)	67 (52-77)	0.199
Age >60 years, n (%)	265 (60)	101 (64)	0.391
Co-morbidities			
Diabetes mellitus, n (%)	83 (19)	39 (25)	0.134
Obesity (body mass index ≥30 kg/m^2), n (%)	73 (17)	29 (19)	0.622
Chronic kidney disease (moderate or severe), n (%)	98 (22)	35 (22)	1.000
Malignancy (solid organ or hematologic), n (%)	55 (13)	22 (14)	0.677
Immunosuppression, n (%)[a]	29 (7)	11 (7)	0.854
Chronic obstructive pulmonary disease, n (%)	41 (9)	14 (9)	1.000
Cirrhosis, n (%)	54 (12)	19 (12)	1.000
Congestive heart failure, n (%)	68 (16)	19 (12)	0.357
Intravenous drug use, n (%)	33 (8)	12 (8)	1.000
Prior infective endocarditis, n (%)	48 (11)	18 (12)	0.882

Surgical prosthetic valve or TAVI, n (%)	122 (28)	51 (33)	0.262
Cardiac implantable electronic device, n (%)	93 (21)	25 (16)	0.199
Charlson Comorbidity Index (points), median (IQR)	3 (1-5)	3 (1-5)	0.229
Charlson Comorbidity Index >4 points, n (%)	143 (33)	57 (36)	0.431
Hospital			0.304
Lausanne University Hospital, n (%)	254 (58)	83 (53)	
University Hospital Zurich, n (%)	186 (42)	74 (47)	
Setting of bacteremia onset			0.538
Community, n (%)	353 (80)	130 (83)	
Healthcare-associated, n (%)	43 (10)	16 (10)	
Nosocomial, n (%)	44 (10)	11 (7)	
Microbiological data			
Three or more positive blood culture sets, n (%)	102 (23)	44 (28)	0.235
Pathogens			
S. aureus, n (%)	164 (37)	71 (45)	0.087
Coagulase-negative staphylococci, n (%)	131 (30)	54 (34)	0.315
Streptococci, n (%)	33 (8)	17 (11)	0.239
Enterococci, n (%)	148 (34)	54 (34)	0.922
Other Gram-positive, n (%)	89 (20)	24 (15)	0.193

HACEK, n (%)	16 (4)	3 (2)	0.428
Gram-negative other than HACEK, n (%)	19 (4)	2 (1)	0.082
Polymicrobial bacteremia, n (%)	12 (3)	6 (4)	0.586
Microbiological data	14 (3)	7 (5)	0.455
Persistent bacteremia for at least 48h from first positive blood culture, n (%)	119 (27)	61 (39)	0.006
Infection data at bacteremia onset			
Fever, n (%)	356 (81)	131 (83)	0.549
Embolic events, n (%)	113 (26)	58 (37)	0.010
Cerebral embolic events, n (%)	63 (14)	24 (15)	0.793
Bone and joint infection, n (%)	47 (11)	15 (10)	0.762
Site of infective endocarditis			
Valve endocarditis, n (%)	392 (89)	152 (97)	0.003
Aortic valve, n (%)	215 (49)	83 (53)	0.404
Mitral valve, n (%)	175 (40)	74 (47)	0.110
Tricuspid valve, n (%)	30 (7)	13 (8)	0.590
Pulmonary valve, n (%)	13 (3)	2 (1)	0.375
Cardiac implantable electronic device -lead, n (%)	59 (13)	11 (7)	0.031
Type of valve			
Native, n (%)	297 (68)	112 (71)	0.424

Prosthetic, n (%)	108 (25)	48 (31)	0.140
Intracardiac lesions			
Vegetation, n (%)	295 (67)	118 (75)	0.070
Vegetation ≥10mm, n (%)	150 (34)	71 (45)	0.016
Abscess, n (%)	65 (15)	34 (22)	0.060
Other lesions, n (%)[b]	54 (12)	22 (14)	0.578
Management			
Source control			0.362
Not warranted, n (%)	330 (75)	125 (80)	
Warranted and performed within 48h, n (%)	21 (5)	4 (3)	
Warranted and not performed within 48h, n (%)	89 (20)	28 (18)	
Antimicrobial treatment			
Timing of antimicrobial treatment initiation			0.304
Group I: immediate initiation, n (%)	235 (53)	92 (59)	
Group D: deferring after blood culture result, n (%)	205 (47)	65 (41)	
Appropriate antimicrobial treatment within 48h, n (%)	418 (95)	146 (93)	0.415

[a]ongoing immunosuppressive treatment at bacteremia onset, intravenous chemotherapy in the 30 days prior to bacteremia onset, or AIDS.

[b]perforation, dehiscence of prosthesis, fistula, aneurysm, pseudoaneurysm.

IQR: interquartile range; Group I: empiric antimicrobial treatment was initiated immediately after the blood culture collection; Group D: antimicrobial treatment was deferred until the first result of blood cultures; TAVI: transcatheter aortic valve implantation

Supplementary Table 7. Univariable and multivariable Cox proportional hazard regression of 30-day primary endpoint (composite of mortality, new embolic event, new bone and joint infection) among 597 infected endocarditis episodes

	Univariable analysis		Multivariable Cox regression	
	P	**HR (95% CI)**	***P***	**aHR (95% CI)**
Staphylococci	0.076	1.32 (0.97-1.80)	0.283	1.22 (0.85-1.76)
Hospital				
Lausanne University Hospital	reference		reference	
University Hospital Zurich	0.227	1.21 (0.89-1.65)	0.042	1.39 (1.01-1.93)
Persistent bacteremia for ≥48 hours from bacteremia onset	0.005	1.58 (1.15-2.18)	0.035	1.50 (1.03-2.18)
Embolic events at bacteremia onset	0.005	1.58 (1.15-2.18)	0.090	1.39 (1.03-2.18)
Valve related endocarditis	0.007	3.33 (1.38-8.02)	0.005	3.56 (1.46-8.68)
Vegetation ≥10mm	0.007	1.54 (1.13-2.10)	0.142	1.31 (0.91-1.86)
Intracardiac abscess	0.052	1.45 (0.99-2.11)	0.379	1.20 (0.80-1.80)
Immediate initiation of antimicrobial treatment	0.268	1.20 (0.87-1.64)	0.093	1.32 (0.95-1.83)

CI: confidence interval; aHR: adjusted hazard ratio

Supplementary Table 8. Univariable and multivariable Cox proportional hazard regression of 30-day mortality among 597 infected endocarditis episodes

	**Univariable analysis**		**Multivariable Cox regression**	
	P	**HR (95% CI)**	***P***	**aHR (95% CI)**
Charlson Comorbidity Index >4	0.001	4.32 (1.86-10.00)	0.001	3.99 (1.70-9.35)
Hospital				
Lausanne University Hospital	reference		reference	
University Hospital Zurich	0.115	0.49 (0.21-1.18)	0.426	0.69 (0.28-1.70)
Persistent bacteremia for ≥48 hours from bacteremia onset	0.002	3.56 (160-7.91)	0.001	4.13 (1.77-9.63)
Appropriate antimicrobial treatment within 48 hours	0.024	0.30 (0.10-0.85)	0.010	0.23 (0.07-0.70)
Immediate initiation of antimicrobial treatment	0.179	1.78 (0.77-4.10)	0.070	2.51 (0.93-6.78)

CI: confidence interval; aHR: adjusted hazard ratio
